# Deceased Organ Donor Management and Organ Distribution From Organ Procurement Organization-Based Recovery Facilities Versus Acute-Care Hospitals

**DOI:** 10.1177/15269248231212918

**Published:** 2023-11-09

**Authors:** Emily A. Vail, Douglas E. Schaubel, Vishnu S. Potluri, Peter L. Abt, Niels D. Martin, Peter P. Reese, Mark D. Neuman

**Affiliations:** 1 Department of Anesthesiology and Critical Care, 14640 University of Pennsylvania, Perelman School of Medicine, Philadelphia, PA, USA; 2 43358 Leonard Davis Institute of Health Economics, University of Pennsylvania, Philadelphia, PA, USA; 3 Penn Center for Perioperative Outcomes Research and Transformation, 14640 University of Pennsylvania, Perelman School of Medicine, Philadelphia, PA, USA; 4 Department of Biostatistics, Epidemiology, and Informatics, 14640 University of Pennsylvania, Perelman School of Medicine, Blockley Hall, Philadelphia, PA, USA; 5 Renal-Electrolyte and Hypertension Division, Department of Medicine, 14640 University of Pennsylvania, Perelman School of Medicine, Philadelphia, PA, USA; 6 Penn Transplant Institute, Philadelphia, PA, USA; 7 Division of Transplantation, Department of Surgery, 14640 University of Pennsylvania, Perelman School of Medicine, Philadelphia, PA, USA; 8 Department of Surgery, 14640 University of Pennsylvania, Perelman School of Medicine, Philadelphia, PA, USA

**Keywords:** research, quantitative methods, regression, procurement, donor maintenance, procurement, organ, donor care units, ischemic time

## Abstract

**Introduction:** Organ recovery facilities address the logistical challenges of hospital-based deceased organ donor management. While more organs are transplanted from donors in facilities, differences in donor management and donation processes are not fully characterized. **Research Question:** Does deceased donor management and organ transport distance differ between organ procurement organization (OPO)-based recovery facilities versus hospitals? **Design:** Retrospective analysis of Organ Procurement and Transplant Network data, including adults after brain death in 10 procurement regions (April 2017-June 2021). The primary outcomes were ischemic times of transplanted hearts, kidneys, livers, and lungs. Secondary outcomes included transport distances (between the facility or hospital and the transplant program) for each transplanted organ. **Results:** Among 5010 deceased donors, 51.7% underwent recovery in an OPO-based recovery facility. After adjustment for recipient and system factors, mean differences in ischemic times of any transplanted organ were not significantly different between donors in facilities and hospitals. Transplanted hearts recovered from donors in facilities were transported further than hearts from hospital donors (median 255 mi [IQR 27, 475] versus 174 [IQR 42, 365], *P* = .002); transport distances for livers and kidneys were significantly shorter (*P* < .001 for both). **Conclusion:** Organ recovery procedures performed in OPO-based recovery facilities were not associated with differences in ischemic times in transplanted organs from organs recovered in hospitals, but differences in organ transport distances exist. Further work is needed to determine whether other observed differences in donor management and organ distribution meaningfully impact donation and transplantation outcomes.

## Introduction/Background

In light of the evidence of superior outcomes among patients who receive care in high-volume surgical centers^1^ and documented challenges and variation in the management and recovery of organs from deceased donors in acute-care hospitals,^2,3^ organ donation in the United States has been increasingly centralized. Over the past two decades, organ procurement organizations (OPOs) have transferred potential donors to other acute-care hospitals with more clinical resources (e.g. available coronary catheterization laboratories or operating rooms), or dedicated organ recovery facilities colocated with OPO offices or within acute-care hospitals.^4^

Rapid facility opening^4,5^ and model endorsement by a recent National Academies of Science, Engineering, and Medicine report^6^ were based on early studies describing operational advantages and improved short-term donation outcomes (as measured by the number of organs transplanted per donor) from a single established OPO-based recovery facility.^7–9^ However, those studies could not ascertain whether local benefits of OPO-facility use could be extrapolated to other regions and OPOs. To address the question of generalizability, a recent retrospective cohort study of 5010 adult deceased organ donors after brain death managed in 10 U.S. OPO regions demonstrated that donors transferred to OPO-based organ recovery facilities donated more transplanted organs on average than donors who remained in local acute-care hospitals.^10^ In that study, secondary donation outcomes (including the proportion of donors with 4 or more organs transplanted, and the numbers of lungs, livers, and pancreases transplanted) were also higher among the group of donors transferred from local hospitals to an available OPO-based facility.

While reasons for observed improvements in the number of organs transplanted from donors who underwent recovery procedures in facilities are likely multifactorial, previously reported differences in donor management processes (including shorter donor management and ischemic times for some organ types) between one OPO-based recovery facility and local hospitals may contribute.^11,12^ As the use of organ recovery facilities accelerates, direct comparisons of donor management between traditional donor processes in acute-care hospitals and OPO-based recovery facilities are needed to inform policymakers,^13^ regulatory agencies,^14^ and other donation system stakeholders.

### Study Aim

This retrospective study compared ischemic times of transplanted organs, donor management and organ assessment processes, and organ distribution distances between donors and organs recovered in OPO-based facilities versus hospitals in 10 U.S. donor regions.

## Design/Methods

### Design

The study was a retrospective comparative analysis of preexisting data captured in U.S. national organ donor and transplant recipient outcomes datasets. The study protocol was reviewed and determined exempt from human subjects’ research by the investigator's Institutional Review Board.

### Population

Between April 2017 and June 2021 (the study period), 45 406 deceased organ donors 18 years or older underwent recovery procedures; donation was most common after brain death (34 688, 76.4% of donors). Among the 11 083 deceased donors managed by the 10 OPOs operating OPO-based recovery facilities (for all or part of the study period), the average age was 41.0 years (standard deviation [SD] 16.3) and 6779 donors (61.2%) were male. Intracranial hemorrhage or stroke was the most common mechanism of death (3035 donors, 27.4%).

### Sampling

A population-based sample of all adult deceased donors after brain death was captured in the study dataset between April 2017 (when organ recovery locations were first recorded) and June 2021 (when analyses were initiated). The cohort was restricted to donors who underwent recovery procedures in an OPO-based facility or regional acute-care hospital on or after the date of facility opening detectable in the study dataset. Donors transferred to facilities were identified using two variables: organ recovery location (classified as Donor Hospital or Alternate Facility) and, for donors in facilities, the facility name (as entered by coordinators in a free text field). Facility names were cross-referenced with publicly available information about operating OPO-based facilities as previously described.^10^ Donors with recovery dates before local facility opening, donors with missing or ambiguous recovery locations (e.g. see coordinator) or donors classified in Alternate Facilities with the name or address of an acute-care hospital were excluded.

### Data Collection

All study data, including organ donor characteristics and short-term transplant recipient outcomes data, were collected by organ procurement and transplant coordinators during routine donor identification, management, organ recovery and transplantation operations; these data were collated, stored, and distributed by the Organ Transplantation and Procurement Network (OPTN) in deceased donor registry and standard transplant recipient files.^15^

### Study Variables

The exposure of interest was organ recovery in an OPO-based organ recovery facility (compared to recovery in an acute-care hospital) as previously described.^10^ Other study variables included transplant recipient and donation system factors plausibly associated with the accrual of ischemic time of transplanted organs, including the year of each organ recovery, recipient body mass index, prior transplant or other surgery likely to impact surgical complexity and duration, type of organ transplant (double or single lung, or whole or partial liver), the hospital of admission of each donor, the transplant program of each transplant recipient, the distance between the organ recovery facility or donor hospital and transplant hospital, and managing OPO. Kidney donor profile and liver donor risk indices were calculated to describe kidney and liver donor characteristics from OPTN data.^16,17^

### Outcomes

As organ ischemic time has been associated with transplantation outcomes,^16,18–20^ the primary outcome of interest was an organ-specific ischemic time (from the application of aortic cross-clamp to reperfusion in the recipient) for each type of organ transplanted with data captured in the study dataset (total ischemic times for hearts and lungs, and cold ischemic times for kidneys and livers, ischemic times hereafter). Times measured from multivisceral transplants (e.g. combined-heart lung and simultaneous pancreas-kidney transplants) were excluded; only the minimum ischemic time (of the first lung implanted) was considered for double lung transplant recipients.

Secondary donor management and organ assessment process outcomes included: (a) organ transport distance (the geodetic distance between the donor hospital or facility and transplant center, calculated from ZIP code centroids, in miles, using the SAS 9.4 ZIPDISTANCE function) for each transplanted organ, (b) time elapsed between hospital admission and diagnosis of brain death diagnosis (in days, limited to the group of donors with a duration of hospital admission 30 days or shorter), and between brain death and organ recovery (in hours), (c) cardiac arrest after diagnosis of brain death, (d) administration of red blood cells and donor-specific medications (including steroids, levothyroxine, and vasopressin, as defined by the United Network for Organ Sharing deceased donor registration worksheet^21^) before organ recovery, (e) kidney and liver biopsies, and (f) the use of ex vivo perfusion for each organ.

### Data Analysis

Each outcome measure was compared between donors who underwent organ recovery in an OPO-based facility and those who remained in hospitals using standard descriptive and comparative statistics. For the primary outcome, grafts with ischemic times below the first and above the 99th percentile for that organ type (determined separately for kidneys that underwent ex vivo perfusion and recipients of single and double lung transplants) were imputed at the first or 99th percentile, respectively; missing values were minimal and therefore not imputed. Missing data were minimal for study variables of interest. Variables with more than 10% of missing values among cohort patients were not reported; Ns for variables with smaller degrees of missingness are reported throughout. Models were restricted to complete cases (donors not missing any model covariates). Except for kidneys (for which ex vivo perfusion was considered in stratified analyses, and as a multivariable model covariate), ischemic times of transplanted organs supported by ex vivo perfusion were excluded from analyses.

For each organ type transplanted, the adjusted mean difference in ischemic times between organ recovery facilities and hospitals was estimated using generalized estimating equations with a linear link function (which does not assume a normal distribution of the outcome). Model covariates considered included donation and transplantation system factors plausibly associated with accrual of ischemic time, including individual donor hospital and transplant program, the distance between recovery facility (for donors recovered in facilities) or donor hospital and transplant program, managing OPO, and type of organ distribution (local, regional, or national as defined by OPTN). Organ recovery year was included as an additional covariate to account for changes in organ allocation policy and transplantation practice over time. Recipient-specific factors that may prolong transplant surgery time (including body mass index, type of transplant, reoperation, repeat transplant, and use of mechanical cardiac circulatory support) were considered where clinically relevant. For models examining organs that may be divided between 2 recipients (kidney, lung, and liver), the type of transplant (e.g. single vs double lung, whole vs partial liver) was also considered for model inclusion. Ex vivo machine perfusion, which may be used to reduce ischemic times during periods of prolonged transport or to further assess the suitability of marginal organs for transplant after recovery,^22^ was considered an additional covariate in the kidney model. Except for the organ distribution region, which was collinear with the calculated distance between the facility or donor hospital and transplant program (and less used in contemporary allocation rules), all covariates with *P* values < .05 in unadjusted analyses were incorporated into organ-specific models. Confidence intervals were adjusted to account for the clustering of paired or split organs from individual donors where applicable.

Secondary donor management and process outcomes between donors in recovery facilities and those in hospitals were compared using descriptive statistics and simple comparison tests without adjustment. Statistical significance was defined as a *P* value of less than .05. Analyses were performed with SAS 9.4 software (Cary, NC). Figures were generated using Microsoft Excel 2016 (Redmond, WA).

## Results

The analysis cohort included 5010 deceased donors after brain death; 2590 (51.7%) donors were transferred from 359 different referring hospitals and underwent recovery procedures in 1 of 10 OPO-based facilities. The remaining donors underwent recovery in 308 acute-care hospitals. Age and sex did not differ between donors in facilities and those in hospitals (**Table 1** and previously described).^10^ Donors in facilities and acute-care hospitals had similar mean kidney donor profile indices (*P *= .16) and left ventricular ejection fractions (*P *= .13); liver donors in facilities had slightly lower donor risk indices^16^ than those in hospitals (*P = *.03). Donors in facilities had higher PaO_2_:FiO_2_ ratios measured before recovery than those in acute-care hospitals 189mmH (IQR 115, 143) versus 155mm Hg [109,313] (*P *< .001).

**Table 1.** Cohort Donor Characteristics.

Organ recovery procedure location	Organ procurement organization-based organ recovery facility	Acute-care hospital	*P* value
	N (%)	N (%)	
Donors	2590 (51.7)	2420 (48.3)	
	Median [Range]	Median [Range]	
Number of donors in each hospital^a^	3 [1-116]	3 [1-197]	
Number of donors in each facility	221 [62-654]	N/A	
	Mean (SD)	Mean (SD)	
Age at organ recovery procedure, years	43.1 (14.5)	43.2 (15.3)	.66
	N (%)	N (%)	
Sex, female	1059 (40.9)	948 (39.2)	.22
Race^b^			
White	1770 (68.3)	1372 (56.7)	<.001
Black	527 (20.4)	405 (16.7)	
Hispanic	236 (9.1)	523 (21.6)	
Asian	38 (1.5)	81 (3.4)	
Other or unknown	19 (0.7)	39 (1.6)	
Organ risk and function indicators			
Kidney donor profile index^b^	Mean (SD)	Mean (SD)	
All donors	0.50 (0.30)	0.51 (0.31)	.16
Donors with kidney(s) recovered for transplant	0.48 (0.29)	0.49 (0.30)	.06
Liver donor risk index^c^	1.53 (0.53)	1.57 (0.55)	.03
PaO_2_: FiO_2_ ratio, median (IQR)	Median [IQR]	Median [IQR]	
All donors	189 [115, 413]	155 [109, 313]	<.001
Donors with lung(s) recovered for transplant	439 [351, 500]	351 [170, 451]	<.001
Left ventricular ejection fraction,^d^ %	60 [56, 65]	60 [57, 65]	.13

Abbreviations: N/A, not applicable; OPO, organ procurement organization; OPTN, Organ Procurement and Transplantation Network.

^a^ For donors in facilities, number of donors transferred from each of 359 referring hospitals. Remaining donors were underwent organ recovery in 308 acute-care hospitals.

^b^ As defined by OPTN.^17^

^c^ As defined by Feng et al^16^ restricted to donors with whole, partial or split livers transplanted, N = 3703.

^d^ Restricted to donors with hearts recovered for transplant, N = 1828.

Nearly all cohort donors (4748, 94.8%) donated at least 1 transplanted organ; 16 517 whole or partial organs were transplanted, with an average of 3.3 (SD 1.8) from each donor. Livers were the most commonly transplanted organ type (4108 donors [82.0% of cohort], 4125 recipients), followed by kidneys (3828 donors [76.4% of cohort], 6811 recipients [alone or with simultaneous pancreas transplants]), hearts (1814 donors [36.1% of donors], 1793 recipients), and lungs (1504 donors [30.0% of cohort], 1569 recipients). Only 442 pancreases (8.8%) and 24 intestines (0.48%) were transplanted from cohort donors.

### Ischemic Times

**Figure 1.**
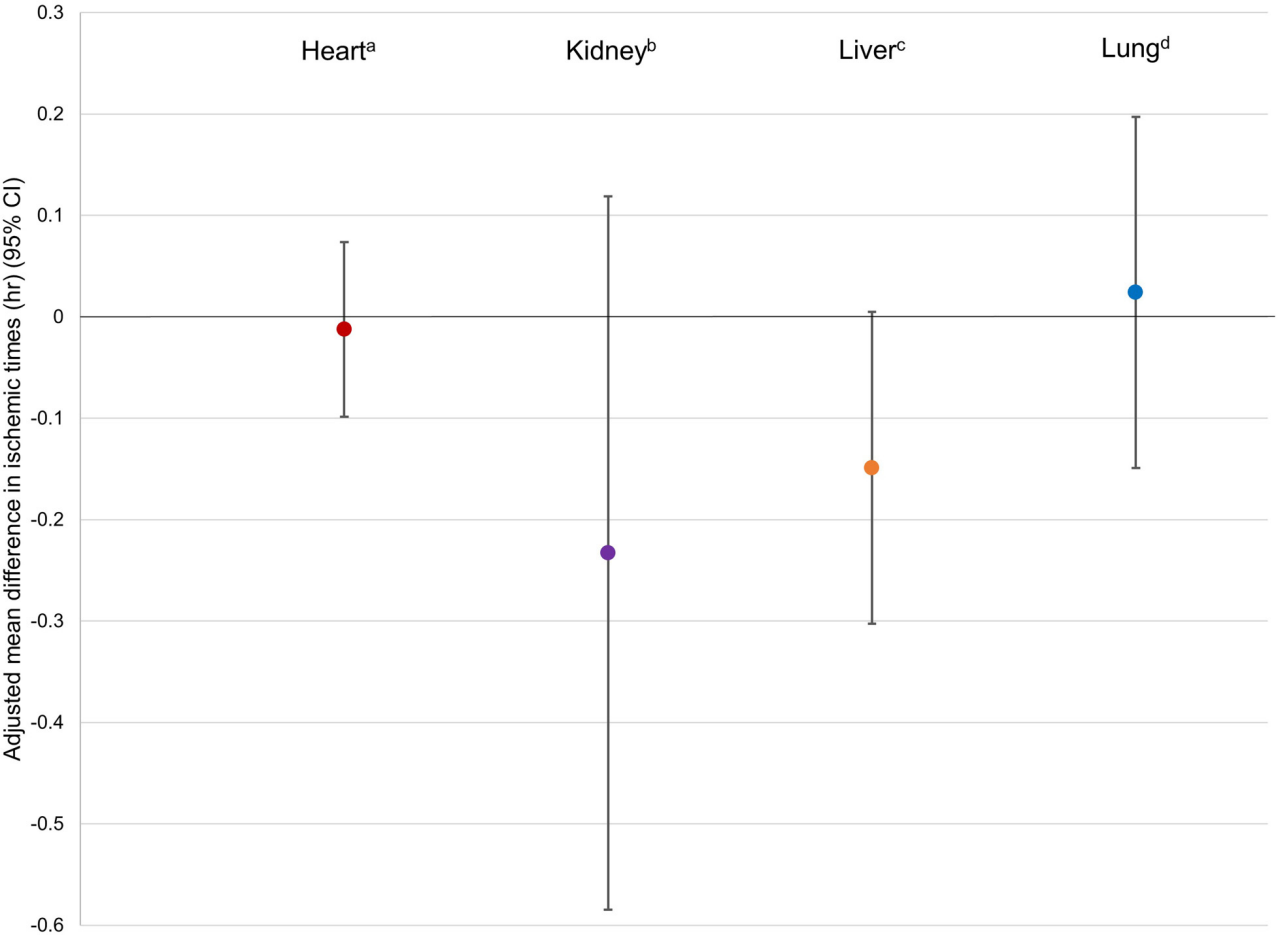
Adjusted mean differences in ischemic times between OPO-based recovery facilities and donor hospitals by type of organ transplanted.

Ischemic times were recorded in 3941 transplanted livers (95.0% of recipients), 6558 (96.3%) transplanted kidneys, 1712 (95.5%) transplanted hearts, and 1495 (95.2%) transplanted lungs. Transplanted livers recovered from donors in OPO-based organ recovery facilities had significantly shorter median ischemic times than those recovered from donors in hospitals (5.4 h; interquartile range [IQR] 4.2, 6.7; vs 6.0 h [IQR 4.8, 7.4], *P* < .001). Median cold ischemic times of kidneys recovered in facilities were significantly shorter than those from donors in hospitals for both kidneys supported by ex vivo perfusion (median 17.7 h [IQR 11.4, 22.8] vs 19.0 h [IQR 14.0, 24.6], *P *< .001) and those without (14.5 h [IQR 8.8, 21.1] vs 15.8 h [IQR 11.1, 21.2], *P *< .001). Unadjusted ischemic times were not meaningfully different for transplanted hearts (median 3.3 h [IQR 2.6, 3.9] in facilities vs median 3.2 h [IQR 2.6, 3.8], *P *= .04) in hospitals, or lungs (5.1 h [IQR 4.2, 6.2] in facilities vs 5.2 h [IQR 4.2, 6.1] in hospitals, *P = *.82). After adjustment, mean differences in ischemic times were not shorter among any organs transplanted from donors in facilities; livers (−0.15 h, 95% confidence interval (CI) −0.30, 0.004), kidneys (−0.23, 95% CI [−0.59, 0.12]), hearts (−0.01, 95% CI [−0.10, −0.07]), or lungs (0.02, 95% CI [−0.15, 0.20]) (**Figure 1**).

All models adjusted for year of organ recovery, managing OPO, donor hospital, transplant program, and distance between OPO-based facility (for donors in facilities) or donor hospital and transplant program.

^a^Total ischemic times. Limited to hearts not supported by ex vivo perfusion or transplanted en block with lungs. Model also adjusts for recipient history of cardiac surgery and receipt of extracorporeal membrane oxygenation or intraaortic balloon pump at the time of transplant.

^b^Cold ischemic times. Excludes kidneys transplanted simultaneously with other organs and kidneys in which the use of ex vivo perfusion was not recorded. The use of ex vivo perfusion (reference group, no ex vivo) and recipient body mass index were included as additional covariates. Times for each transplanted graft are included separately in the model with confidence intervals adjusted to account for clustering by donor.

^c^Cold ischemic times. Excludes livers supported by ex vivo machine perfusion. Whole (ref.) versus split or partial liver transplant, recipient model for end-stage liver disease (MELD) score at time of transplant, and recipient history of upper abdominal surgery (ref. no surgery) included as additional covariates in the multivariable model. Times for each transplanted graft are included separately in the model with confidence intervals adjusted to account for clustering by donor.

^d^Total ischemic times. Excludes lungs transplanted simultaneously with hearts and lungs supported by ex vivo perfusion. Model also includes single lung transplant (ref.) versus double, recipient receipt of extracorporeal membrane oxygenation at the time of transplant, and recipient body mass index as covariates. Times for each transplanted graft are included separately in the model with confidence intervals adjusted to account for clustering by donor.

The median number of days from hospital admission to organ recovery (5 days [range 1-29)] vs 6 days [1-28], *P *< .001), and the median number of hours elapsed between brain death diagnosis and organ recovery (47 h [range 8-153] vs 51 h [range 8-152], *P *< .001) were shorter among donors managed in OPO-based organ recovery facilities (**Table 2**). Donors in recovery facilities were significantly more likely to suffer cardiac arrest after diagnosis of brain death (323 donors [12.7%] in facilities vs 209 [8.9%] in hospitals, *P *< .001 for the difference, without adjustment). Donors transferred to recovery facilities were significantly less likely to receive levothyroxine (35.2% vs 51.5%, *P *< .001) and vasopressin (50.6% vs 58.2%, *P *< .001) after brain death than donors that remained in local hospitals. The incidences of kidney and liver biopsies were not significantly different between facilities and hospitals (*P = *.43 and *P = *.60, respectively), but rates of ex vivo perfusion of recovered kidneys and livers were significantly higher among organs recovered from donors in OPO-based facilities than in hospitals (*P *< .001 and *P *= .003, respectively); use of ex vivo lung perfusion did not differ between groups (*P *= .84) and was rare for recovered hearts.

**Table 2.** Differences in Clinical Donor Management and Organ Assessment Processes Between Donors Managed in Organ Procurement Organization-Based Organ Recovery Facilities Versus Those in Hospitals.

Organ recovery procedure location	Organ procurement organization-based organ recovery facility	Acute-care hospital	*P* value
	N (%)	N (%)	
Donors	2590 (51.7)	2420 (48.3)	
Duration of donor management			
	Median (range)	Median (range)	
Days between acute-care hospital admission and organ recovery procedure, whole or partial calendar days^a^	5 (1-29)	6 (1-28)	<.001
Hours between brain death diagnosis and organ recovery procedure^a^	47 (8-153)	51 (8-152)	<.001
	N (%)	N (%)	
Cardiac arrest after declaration of brain death	323 (12.7)	209 (8.9)	<.001
Clinical donor management	Median (range)	Median (range)	
Number of red blood cell transfusions (units)^b^	0 (0-3)	0 (0-3)	<.001
	N (%)	N (%)	
Received levothyroxine^b^	912 (35.2)	1236 (51.1)	<.001
Received arginine vasopressin^b^	1310 (50.6)	1408 (58.2)	<.001
Received inotropic or vasoactive medications at time of aortic cross clamp	1377 (53.2)	1261 (52.1)	.85
Diagnostic procedures to assess organ quality			
Organ biopsies			
Kidney	1287 (49.7)	1227 (50.7)	.43
Liver	1181 (45.6)	1075 (44.4)	.60
Interventions for recovered organs			
Ex vivo perfusion			
Kidney^c^	1136 (48.5)	619 (28.1)	<.001
Liver^d^	41 (1.8)	16 (0.8)	.003
Lung^e^	48 (5.6)	40 (5.8)	.84
Heart^f^	9 (0.9)	4 (0.5)	.19

^a^ Limited to the group of donors with hospital admissions of 30 or fewer whole or partial calendar days.

^b^ Administered in hospital of admission or OPO-based donor management facility.

^c^ At least one donated kidney supported with ex vivo perfusion. Limited to donors with at least one kidney recovered for transplant.

^d^ Among donors with whole or partial liver recovered for transplant.

^e^ At least one donated lung supported with ex vivo perfusion. Limited to donors with one or both lungs recovered for transplant.

^f^ Among donors with heart recovered for transplant.

**Figure 2.**
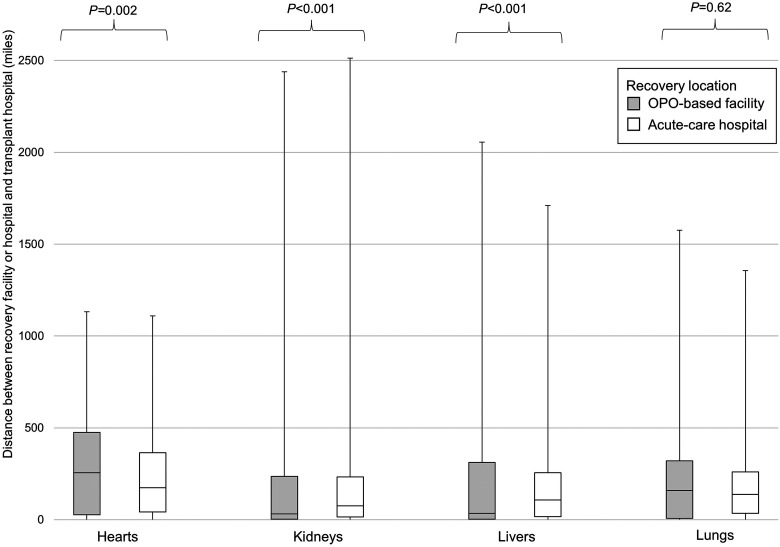
Transport distances of organs from deceased donors in organ procurement organization-based recovery facilities versus acute-care hospitals.

Transport distances differed between organs recovered in facilities and those in hospitals and varied by type of transplanted organ (**Figure 2**). Median transport distance was higher among hearts recovered in facilities (255 geodesic miles [IQR 27, 475] vs 174 [IQR 42, 365] in hospitals, *P = *.002). However, kidneys and livers recovered from donors in facilities were transported shorter distances than those recovered in hospitals (median 32 geodesic miles [IQR 3, 236] vs 75] [15, 233] in hospitals, *P *< .001 for kidneys; median 34 [IQR 3, 312] vs 108 [17, 256], *P *< .001 for livers). There was no significant difference in lung transport distances between recovery locations (*P *= .62).

Distances calculated between ZIP code centroids of OPO-based recovery facilities and transplant program hospitals (for donors who underwent recovery procedures in facilities) and between donor hospitals and transplant hospitals (for donors who remained in acute-care hospitals). *P* values represent unadjusted comparisons between donors in facilities versus hospitals for each organ type.

## Discussion

In a retrospective cohort analysis of 5010 deceased organ donors after brain death managed by 10 U.S. OPOs, ischemic times of organs recovered from donors in OPO-based recovery facilities were not different from those of organs recovered in hospitals. The study identified differences in clinical donor management and support processes between recovery locations, including shorter donor management times, the use of specific medications during donor management, the use of ex vivo perfusion of recovered kidneys and livers, and differences in organ transport distances for hearts, livers, and kidneys.

While prior analyses of donation outcomes using the same study cohort and exposure of interest found slightly but significantly higher numbers of organs transplanted from donors who underwent recovery procedures in OPO-based facilities,^10^ results of the current study do not clearly indicate which factor or factors may be responsible for that observed difference. Instead, results illustrate the complex interplay of donor characteristics, clinical management, and transplant program practices that may impact the likelihood of donor transfer to a facility, and the numbers and types of organs considered and accepted for transplant, and subsequently recovered and transplanted from each donor.

Study findings were consistent with, and expanded upon, previous single-center work, which demonstrated shorter donor management times for donors transferred to recovery facilities.^11,23^ Past studies also demonstrated shorter unadjusted ischemic times of some organs recovered in facilities (vs local hospitals) with specific patterns of distribution (e.g. for livers distributed regionally, but not locally).^11,12^ While shorter unadjusted ischemic times for transplanted organs from donors in recovery facilities have been invoked as potential benefits of OPO-based facility management, results of the present study (which accounts for recipient factors and organ transport distance) challenge this presumption and may call into question whether the widespread opening of recovery facilities has outpaced the strength of available evidence. As facility opening requires significant financial and human resource investments, further work is needed to determine whether differences in longer-term, patient-centered outcomes, such as time on waitlists, the likelihood of transplant, and the duration of survival after transplant, exist between organs recovered in facilities versus hospitals and may justify their adoption nationwide.

Organ transport distance differences between donors in facilities versus hospitals observed in this study were not directly comparable to higher local rates transplantation rates reported in the first few years of operation of one OPO-based facility opening,^11^ but were consistent with shorter distances traveled for kidneys and livers recovered in facilities.^11^ Because more cohort donors underwent recoveries in facilities over time,^10^ observed differences may reflect changes in specific organ allocation rules during the study period^19^ or greater willingness of distant transplant programs to travel to OPO-based facilities located near major airports (requiring less travel time than to reach more remote donor hospitals within the same OPO region).^24^ It is not yet known whether organ recovery facilities may mitigate or exacerbate known geographic disparities in access to transplantation within or across donor service areas and UNOS regions.^25^

Differences in donor management between facilities and hospitals observed in this study may reflect differences in clinical protocols used to guide donor management between locations,^26^ differences in adherence to those protocols, or in some cases, incentives for OPOs to reduce donor management costs by reducing the use of expensive therapies when cheaper alternatives are available (e.g. replacing vasopressin with norepinephrine) or expediting organ recovery (to decrease staffing costs accrued during longer periods of donor management) that may be easier to achieve in facilities. Other observed differences may reflect specific donor management resources that may be more available or frequently used at OPO-based facilities, such as ex vivo perfusion equipment. However, as analyses were retrospective and did not include measures of donor severity of illness, the possibility that observed differences rose from confounding by indicationd out cannot be rule.

Despite shorter management times among donors who underwent recovery procedures in facilities, donors in facilities had significantly higher rates of cardiac arrest after brain death than donors remaining in acute-care hospitals. While donors in facilities and hospitals were similar in most clinical characteristics, and equally likely to receive vasopressors or inotropes in the 24-h period before recovery procedures (suggesting that the donors had similar hemodynamic profiles between groups), without available data on the time, location, and outcomes of these events, the risk of cardiac arrest or other adverse events during transport to and management in recovery facilities remains a concern. An alternate explanation is that donors who suffer cardiac arrest in hospitals may be less likely to be resuscitated until the return of spontaneous circulation or considered for donation and recovery after arrest than those in facilities. Given that the clinical decompensation of potential donors threatens the availability and quality of organs for transplant, future studies of deceased organ donor management should specifically measure and compare these adverse events and other indicators of donor management quality and facility performance.

Study limitations include those inherent to retrospective analyses of secondary data. In this study more specifically, the study dataset did not contain information about why individual donors were, or were not, transferred to an available facility or specific time stamps for donor transfer, therapy administration, or adverse events, potentially misclassifying these in hospitals versus facilities. Analyses of ischemic times could not account for all factors (such as unanticipated transportation delays) that may impact measured ischemic times. Other donor management practices (e.g. tissue biopsies) may be performed by OPOs attempting to allocate higher-risk organs or at the request of transplant programs assessing offered organs and cannot be attributed to decision-making or practices unique to facilities alone. Study data were insufficient to examine whether other reported benefits of a single OPO-based facility (including satisfaction of donor families and recovering surgeons, and satisfaction and retention of OPO staff)^27^ apply to other operating facilities or to examine other factors (such as clinical staffing resources and staff workload) likely to differ between facilities and hospitals.

Future work is needed to understand how diverse OPOs may best use organ recovery facilities to serve heterogeneous populations with varying acute healthcare resources. No studies to date have examined, or answered this question, because the data collected during donor management do not include key factors that inform decisions to open or operate a facility or, when facilities are available, to transfer individual donors.

## Conclusions

In a retrospective study of deceased organ donors after brain death in 10 U.S. OPOs, adjusted ischemic times of organs recovered from donors managed in OPO-based facilities were not different from those recovered in hospitals. Observed differences in secondary donor management practices (including organ travel distance and use of ex-vivo perfusion) require further investigation to determine which differences reflect evidence-based practice and consistently impact donation system performance measures and the donation and transplantation outcomes needed to mitigate the critical organ shortage.
